# Adherence to antiretroviral therapy and associated factors among HIV positive adults attending care and treatment in University of Gondar Referral Hospital, Northwest Ethiopia

**DOI:** 10.1186/s12879-018-3176-8

**Published:** 2018-06-08

**Authors:** Abiyot Abeje Molla, Abebaw Addis Gelagay, Habtamu Sewunet Mekonnen, Destaw Fetene Teshome

**Affiliations:** 1 HIV care and Treatment Clinic, University of Gondar Referral Hospital, Gondar, Ethiopia; 2 Department of Reproductive Health, Institute of Public Health, College of Medicine and Health Sciences, University of Gondar, Gondar, Ethiopia; 3 Department of Medical Nursing, School of Nursing, College of Medicine and Health Sciences, University of Gondar, Gondar, Ethiopia; 4 Department of Epidemiology and Biostatistics, Institute of Public Health, College of Medicine and Health Sciences, University of Gondar, Gondar, Ethiopia

**Keywords:** HIV positive adults, Antiretroviral therapy, Adherence, Ethiopia

## Abstract

**Background:**

Antiretroviral therapy has an impressive clinical effect on the human immunodeficiency virus although its effectiveness depends mainly on the adherence of patients to the therapy. Therefore, this study aimed to assess adherence status and associated factors of antiretroviral therapy among HIV infected adults on ART at the University of Gondar Referral Hospital, northwest Ethiopia.

**Methods:**

An institutional based cross-sectional study was conducted from May to June 2015. The systematic random sampling technique was used to select 440 study participants. Data collected using an intervieweradministered questionnaire was entered using EPI Info version 7 and analyzed using SPSS version 20. Both bivariate and multivariate logistic regression analyses were done. In the multivariate analysis, variables with *P*-value ≤ 0.05 were considered statistically significant between independent variables and the outcome variable (medication adherence). Adjusted odds ratio (AOR) with a 95% confidence interval was used to determine the strength and direction of the association.

**Results:**

A total of 440 participants were included in the study. The mean age of participants was 36.09 (SD ± 8.09) years. The overall rate of adherence to ART was 88.2% (95% CI = 85.2, 91.1). Urban residence (AOR = 6.99, 95% CI: 2.30, 21.27), no co-morbidity (AOR = 0.13, 95% CI: 0.05, 0.33), knowledge about HIV and ART (AOR = 7.54, 95% CI: 2.69, 21.15), and disclosed HIV status to partners (AOR = 3.65 (1.06, 12.61) and CD4 count of ≥ 500mm^3^ (AOR = 3.91, 95% CI: 1.19, 12.81) were significantly associated with adherence.

**Conclusion:**

In this study, the rate of adherence to antiretroviral therapy was low compared to WHO standard.. Prevention of co-morbidities, improving knowledge through health education, providing strong drug adherence counseling with more emphasis on the rural community, and encouraging HIV positive individuals to disclose their HIV status are crucial for ART adherence.

## Background

HIV/AIDS, with which 36.7 million people were living and 2.1 million newly infected at the end of 2016, has been one of the major health problems globally. From the beginning of the epidemic to 2016, 35 million people died from AIDS-related illnesses [1–3]. Although the burden of HIV continues to vary significantly across countries, Sub-Saharan Africa remains the most affected with almost 1 in every 25 adults (4.4%) living with it, accounting for nearly 70% of the global burden [2]. According to the 2014 estimate the national HIV prevalence in Ethiopia was 1.14%, and the number of people living with HIV is 769, 600 with 15, 700 new HIV infections and 35, 600 AIDS-related deaths each year [4].

The Human Immunodeficiency Virus (HIV) infection does not only upset the health of individuals but also impacts on households, communities, and the development of nations. When countries are affected by HIV, they also suffer from other infectious diseases, food insecurity, and other serious problems [5]. Antiretroviral therapy has an impressive clinical effect in that it decreases the viral replication and viral load which in turn preserves the CD4 level, decreases the progress of AIDS, and reduces AIDS-related deaths [6].

However, the clinical outcomes of ART depend mainly on the adherence of patients to antiretroviral therapy. Studies show that adherence to antiretroviral therapy is essential to reduce the multiplication of the virus and improve disease outcomes [7, 8]. they further demonstrate that medication adherence is second to CD4 count for precisely anticipating progression to AIDS and death [9, 10]. All round efforts such as increasing accessibility of HIV screening, counseling, and ART services by private, government and non-government organizations have been made to reduce morbidity and maintain the quality of life of HIV-positive individuals [11]. Studies indicate that > 95% adherence to therapeutic schemes is required for an HIV infected patient to reach full viral suppression [12–15]. However, adherence to therapeutic regimens might be difficult for many reasons. For example, the side effects of HIV medicines could pose challenges on strict adherence. Besides, the medication dosing schedule might not fully the patient’s daily activities [16, 17].

Poor -adherence to highly active antiretroviral therapy (HAART) diminishes the effectiveness of ART and accelerates viral dissemination and drug resistance [18, 19]. This catastrophic event affects patients by reducing their quality of life and the community as well as the health system by increasing medical costs.

There has been a considerable progress of access to ART and HIV counseling and testing in Ethiopia [20]. There are however only a few studies on the adherence status of ART and its determinant factors in various parts of the country. The level of adherence is highly affected by the level of commitment of ART service providers in providing “drug adherence counselling services”. Since this specialized hospital is providing services to more than 5 million people and over five thousand ART users in Amhara Region, knowing the level of ART adherence and its determinants is essential for making appropriate interventions.

Therefore, this study aimed to assess adherence status and associated factors of ART among HIV infected adult patients on ART at the University of Gondar Referral Hospital, northwest Ethiopia.

## Methods

### Study design and setting

An institution based cross-sectional study was conducted from May to June 2015 at the University of Gondar Referral Hospital Chronic HIV Care and Treatment Clinic. The hospital is found in Gondar town, 727 km from Addis Ababa, the capital of Ethiopia. This teaching hospital serves for more than five million people in the zone and nearby districts and started free ART service in 2006. Currently, it has four outpatient, one ART drug refill and pre ART follow up, one voluntary testing and counseling rooms, a pharmacy and a laboratory. More than 7500 adults and 700 pediatric patients were enrolled in the HIV care centre after the hospital started the care. More than 5100 of the adults were on ART at the movement. In Ethiopia, the first- line ART regimen is (TDF + 3TC + EFV), while AZT + 3TC + EFV, AZT + 3TC + NVP, and TDF + 3TC + NVP are used as an alternative. The second line regimen is TDF + 3TC + LPV/r or ATV/r and AZT + 3TC + LPV/r or ATV/r, whereas AZT + TDF + 3TC + (ATV/r or LPV/r) is given to patients with HBV co-infection.

### Source/study participants

All HIV infected adults aged ≥18 years e attending care and treatment the ART Clinic at the University of Gondar Referral Hospital were the study population. However**,** patients who had a hearing problem and known mental disorder were excluded.

### Sample size determination and sampling procedure

The sample size was calculated using the single population proportion formula (n = [(Zα/2)^2^ × P (1-P)]/D^2^) with the assumption of a 95% level of confidence, 3.5% margin of error, 85% rate of adherence to ART detected in Harar and Dire Dawa, eastern Ethiopia [21], and a 10% nonresponse rate which yielded 440. Participants were selected using the systematic random sampling technique, and every 12 patients were interviewed based on their order of arrival.

### Data collection tool and procedure

A structured interviewer-administered questionnaire was used to collect data. The questions on the explanatory variables were prepared using the WHO conceptual model and by reviewing international literature.

Pill count is cost effective, simple, and more accurate than other methods [22]. However, the number of pills left does not necessarily reflect a consistent use of the drugs. Therefore, adherence status was assessed based on the number of pills reported to have been actually taken one month prior to the data collection period divided by the number of prescribed pills multiplied by 100%. Patients who reported an intake of ≥95% of the prescribed medication were considered adherent; those with a reported intake of < 95% were classified as non-adherent.

The questionnaire was first prepared in English and translated to Amharic, the local language, and then retranslated to English to ensure the consistency of the tool. The pre-test was done on 26 HIV infected patients on ART at nearby hospital, and modifications were made based on the findings. One MSc and four BSc degree graduate nurses who were working out of the ART clinic were recruited for supervision and data collection, respectively, after they were trained for two days. The investigators and the supervisor monitored the data collection throughout the process.

### Operational definition

#### Knowledgeable

Those respondents who scored points at mean and above for the knowledge question prepared on HIV and its treatment otherwise not.

#### Comorbidity

The presence of any of the chronic disease along with HIV/AIDs.

### Data processing and analysis

Each item of the questionnaire was checked for completeness and coded manually before data entry. The data were entered using Epi info version 7 and exported to SPSS version 20 for data analysis. Descriptive statistics, including frequencies, means and standard deviations were used to describe the data. Bivariate logistic regression analysis was carried out and variables with *P*-values of ≤ 0.2 were entered into the multivariate logistic regression for final analysis. Hosmer and Lemeshow fitness of Good test was computed. And adjusted odds ratio with a 95% Confidence interval (CI) was computed to see the presence of strength and the direction of association between dependent and independent variables.

## Result

### Socio-demographic characteristics of participants

A total of 440 participants were interviewed with a 100% response rate. The mean age of participants was 36.09 (SD ± 8.09) years. More than half, 264 (60%), of the participants were females, 378 (85.9%) Orthodox Christians and 375 (85.2%) Amhara by ethnicity. The majority, 377 (85.7%), and a quarter, 106 (24.1%), of the participants were urban dwellers and had no formal education, respectively (Table 1).

**Table 1:** Socio-demographic characteristic of HIV positive adults attending care and treatment in University of Gondar Referral Hospital, Northwest Ethiopia, 2015 (*n* = 440)

Variable	Frequency	Percent (%)
Sex		
Male	176	40.0
Female	264	60.0
Age		
18–28	77	17.5
29–38	212	48.2
39–48	119	27.0
> =49	32	7.3
Marital status		
Single	66	15.0
Married	236	53.6
Divorced	69	15.7
Widowed	69	15.7
Religion		
Orthodox	378	85.9
Protestant	15	3.4
Muslim	47	10.7
Ethnicity		
Amhara	375	85.2
Tigrie	25	5.7
Kimant	40	9.1
Residence		
Urban	377	85.7
Rural	63	14.3
Educational status		
No formal education	106	24.1
Primary school	83	18.9
Secondary school	210	47.7
College/University	41	9.3
Occupational status		
Government employee	87	19.8
Private employee	75	17
Housewife	93	21.1
Merchant	86	19.5
Farmer	33	7.5
Daily laborer	26	5.9
Others ^a^	40	9.1
Income ^b^		
< 1000	118	26.8
1000–2000	127	28.9
2001–3000	85	19.3
> 3000	110	25

^a^student, garden

^b^In Ethiopian Birr

### Clinical characteristics of participants

Of the participants, 297 (67.5%) were knowledgeable about HIV/AIDS and its treatments; more than half, 240 (54.5%) knew about the HIV status of their partners, and 200 (45.5%) of the partners were seropositive; the CD4 count of 266 (60.5%) was < 500 mm^3^ (Table 2).

**Table 2:** Clinical characteristics and antiretroviral therapy condition of the study participants, University of Gondar Referral Hospital, Northwest Ethiopia, 2015 (*n* = 440)

Variables	Frequency	Percent
Comorbidities		
Yes	117	26.6
No	323	73.4
Knowledge about treatment		
Knowledgeable	297	67.5
Not knowledgeable	143	32.5
Knowing HIV status of partners		
Yes	240	54.5
No	200	45.5
HIV status of the partner (*n* = 240)		
Positive	200	83.3
Negative	40	16.7
Family disclosure status		
Yes	321	73.0
No	119	27.0
CD4 count		
< 500 mm3	266	60.5
> = 500 mm3	174	39.5
Substance use in the past 1 year		
Yes	29	6.6
No	411	93.4
Partner in the past 1 year		
Yes	314	71.4
No	126	28.6
Length of stay with a current partner (*n* = 314)		
< 1 year	67	21.3
1–4 year	39	12.4
4 year	208	66.3
Number of partners (*n* = 314)		
One	268	85.4
More than one	46	14.6
Discussion about safe sex (n = 314)		
Yes	256	81.5
No	58	18.5
Duration since tested HIV positive		
< 1 year	30	6.8
1–5 year	172	39.1
5–10 year	231	52.5
> 10 years	7	1.6
Duration since on ART		
< 1 year	62	14.1
1–5 years	377	85.7
> 5 years	1	0.2

### The overall level of adherence to antiretroviral therapy

In this study, the rate of adherence to antiretroviral therapy was 88.2% (95% CI = 85.2, 91.1). The rate is higher (92.0%) among urban and lower (65.1%) among rural residents.

### Factors associated with adherence to antiretroviral therapy

The multivariate logistic regression analysis showed that urban residence, absence of co-morbidity, knowledge about HIV/AIDS, and ART, disclosing HIV status to partner and recent CD4 count ≥ 500 mm^3^ were significantly associated with adherence to antiretroviral therapy.

Thus, HIV infected patients who lived in urban settings were 7 times (AOR = 6.99, 95% CI: 2.30, 21.27) more likely to adhere to their antiretroviral therapy (ART) than rural residents. Participants without co-morbidity were 87% (AOR = 0.13; 95% CI: (0.05, 0.33) less likely to adhere than participants with co-morbidity. Furthermore, participants who were knowledgeable about HIV and antiretroviral therapy were 7.5 times (AOR = 7.54 95% CI: 2.69, 21.15) more likely to be adherent compared with non-knowledgeable patients. In addition, participants who disclosed their HIV status to their partners were 3.7 times (AOR = 3.65 (1.06, 12.61)) as likely to be adherent as those who did not disclose. The odds of ART adherence was found high among participants with a CD4 count of ≥ 500mm^3^ (AOR = 3.91 95% CI: 1.19, 12.81)) (Table 3).

**Table 3:** Bivariate and multivariate analysis for adherence to antiretroviral therapy among HIV positive adults attending care and treatment in University of Gondar Referral Hospital, Northwest Ethiopia, 2015 (*n* = 440)

Variables	Adherence Status		COR (95% CI)	AOR (95% CI)
	Adherent	Non Adherent		
Sex				
Male	153	23	1	
Female	235	29	1.22(0.68,2.18)	
Age (in year)				
18–28	66	11	1.11(0.35,3.50)	
29–38	191	21	1.1.68(0.59,4.84)	
39–48	104	15	1.28(0.43,3.85)	
> = 49	27	5	1	
Marital status				
Single	54	12	0.76(0.31,1.91)	
Married	213	23	1.57(0.71,3.48)	
Divorced	62	7	1.50(0.54,4.20)	
Widowed	59	10	1	
Educational status				
No formal education	97	9	1.17(0.34,4.02)	
Primary school	75	8	1.01(0.29,3.58)	
Secondary school	79	31	0.62(0.21,1.88)	
College/University	36	5	1	
Occupation				
Government employee	79	8	3.75(0.97,10.24)	
Private employee	70	5	5.31(0.89,16.64)	
Housewife	85	8	4.03(0.87,10.99)	
Merchant	74	12	2.34(0.93,5.89)	
Farmer	27	6	1.71(0.55,5.25)	
Daily laborer	20	6	4.55(0.92,22.56)	
Others	29	11	1	
Residence				
Urban	347	30	6.21(3.28,11.75)^a^	6.99(2.30,21.26) ^b^
Rural	41	22	1	1
Income^c^				
< 1000	92	26	0.28(0.12,0.64)^a^	0.42(0.12,1.52)
1000–2000	116	11	0.83(0.32, 2.14)	0.95(0.24,3.69)
2001–3000	78	7	0.87(0.30,2.51)	1.09(0.27,4.41)
> 3000	102	8	1	1
Comorbidities				
Yes	82	35	0.13(0.07, 0.24)^a^	0.13(0.05, 0.33)^b^
No	306	17	1	1
Knowledge about treatment				
Knowledgeable	282	15	6.56(3.46,12.45)^a^	7.54(2.69,21.15)^b^
Not Knowledgeable	106	37	1	1
Knowing HIV status of partners				
Yes	220	20	0.33(0.16,0.68)^a^	0.57(0.64,4.96)
No	58	16	1	1
HIV status of partners				
Negative	35	5	0.57(0.19,1.66)	0.91 (0.21, 4.01)
Positive	185	15	1	1
Discuss about safe sex with a partner				
Yes	232	24	0.4(0.19,0.85)^a^	0.83(0.18,3.88)
No	46	12	1	1
Disclosing HIV status of partners				
Yes	369	42	4.62(2.02,10.60)^a^	3.65(1.06,12.61)^b^
No	19	10	1	1
Recent CD4 count				
< 500 mm^3^	227	39	1	1
> =500 mm^3^	161	13	2.13(1.1,4.11)^a^	3.91(1.19,12.81)^b^
Number of partner				
1	240	28	1.81(0.77,4.25)	1.01(0.21,4.73)
> 1	38	8	1	1
Length of stay with current partner				
< 1 year	56	11	0.45(0.20,1.02	2.9(0.47,17.97)
1–4 years	31	8	0.35(0.14,0.87)^a^	0.76(0.19,3.14)
> 4 years	191	17	1	1
Duration since tested HIV positive				
< 1 year	24	6	0.67(0.07,6.64)	
1–5 year	154	18	1.43(0.16,12.52)	
5–10 year	204	27	1.26(0.15,10.86)	
> 10 years	6	1	1	

^a^Significant variables in bivariate analysis,

^b^Significant variables in multivariate analysis,

^c^In Ethiopian Birr

## Discussion

In this study, the rate of adherence to antiretroviral therapy was found to be 88.2% (95% CI = 85.2, 91.1). This is almost in-line with those of studies done at Dessie referral hospital [3], Debre Markos referral hospital [7], Eastern Ethiopia [5], and Global Hospital [10], and Nepal [6], which reported 90, 88.6, 85%, and, 90.8, and 85.5%, respectively.

However, it is higher than there of studies done in Savannakhet and Sethathirath hospitals (60%) [4], Dubti hospital (81.1%) [11], Myanmar (76.24%) [12], China (81.8%) [13], and a Tertiary care hospital in Aurangabad (78%) [1]. This difference might be due to differences in socio-demographic characteristic, sample size large in this study, and difference in study designs.

This finding is lower than that of another study conducted in Debre Birhan referral hospital and health center (95.5%) [9]. The variation might be due to differences in study settings and socio-demographic characteristics.

In the current study, associations were observed between adherence to ART and different variables. Urban residence had a significant association with good adherence. The possible justification could be that in this study urban residents were more educated. This might increase their awareness and access to HIV therapy. The presence of co-morbidity had a significant association with the outcome variable. In the study, participants without co-morbidity had good adherence to antiretroviral therapy, like a similar study reported elsewhere [5]. The possible explanation might be that when patients had co-morbidities, they might have pill burden. As a result, they might miss ART medications as they give more attention to the acute problem.

Being knowledgeable about HIV and antiretroviral therapy had a significant association with good adherence. This is similar to the results of studies done in Nepal [6] and China [13]. Disclosure of status of HIV to family/partner also had a significant association with good adherence. This agreed to the findings of studies done in Dessie referral hospital, eastern Ethiopia, Felege-Hiwot and Gondar University hospitals [3, 5, 8] and Nepal [6].

Recent CD4 count was significantly higher among patients with good ART adherence. This is similar to the findings of a study done in HIV Epidemiology Research (HER) in the US [2].

In this study, adherence was assessed according to data actually taken during the previous one month. So, the participants might be subjected to recall bias. Since the data was collected using an interviewer administered technique, it might also be exposed to social desirability bias. However, the participants were adequately informed about the relevance of the study and the importance of genuineness. Additionally, the data collectors were professionals working out of the ART clinics. Patients attending health centers were not included in this study. This might impose a limitation on the generalization of the findings to all ART users in the region.

## Conclusion

In this study, antiretroviral therapy adherence was found low compared to the WHO standard. Urban residence, no co-morbidity, being knowledgeable about HIV and its treatment, disclosing HIV status to partners and CD4 count ≥ 500 mm^3^ were factors significantly associated with good ART adherence. High CD4 count was found to be a proxy indicator of good ART adherence. Thus, prevention of co-morbidities, improving knowledge through health education, providing strong drug adherence counseling with more emphasis on the rural community, and encouraging HIV positive individuals to disclose their HIV status are crucial for ART adherence.

## Abbreviations

AOR: Adjusted odds ratio
ART: Antiretroviral Therapy
COR: crude odds ratio
HIV/AIDS: Human immune deficiency virus/ Acquired immune-deficiency syndrome
PLWHA: people living with HIV/AIDS
SPSS: a Statistical package for social sciences
